# Desminopathies: pathology and mechanisms

**DOI:** 10.1007/s00401-012-1057-6

**Published:** 2012-11-11

**Authors:** Christoph S. Clemen, Harald Herrmann, Sergei V. Strelkov, Rolf Schröder

**Affiliations:** 1 Institute of Biochemistry I, Medical Faculty, University of Cologne, Joseph-Stelzmann-Street 52, 50931 Cologne, Germany; 2 Functional Architecture of the Cell, German Cancer Research Center (DKFZ), Im Neuenheimer Feld 580, 69120 Heidelberg, Germany; 3 Laboratory for Biocrystallography, Department of Pharmaceutical and Pharmacological Sciences, Katholieke Universiteit Leuven, O&N II Herestraat 49, Box 822, 3000 Leuven, Belgium; 4 Institute of Neuropathology, University Hospital Erlangen, Schwabachanlage 6, 91054 Erlangen, Germany

**Keywords:** Desmin, Desminopathy, Intermediate filaments, Myofibrillar myopathy, Protein aggregate myopathy

## Abstract

The intermediate filament protein desmin is an essential component of the extra-sarcomeric cytoskeleton in muscle cells. This three-dimensional filamentous framework exerts central roles in the structural and functional alignment and anchorage of myofibrils, the positioning of cell organelles and signaling events. Mutations of the human desmin gene on chromosome 2q35 cause autosomal dominant, autosomal recessive, and sporadic myopathies and/or cardiomyopathies with marked phenotypic variability. The disease onset ranges from childhood to late adulthood. The clinical course is progressive and no specific treatment is currently available for this severely disabling disease. The muscle pathology is characterized by desmin-positive protein aggregates and degenerative changes of the myofibrillar apparatus. The molecular pathophysiology of desminopathies is a complex, multilevel issue. In addition to direct effects on the formation and maintenance of the extra-sarcomeric intermediate filament network, mutant desmin affects essential protein interactions, cell signaling cascades, mitochondrial functions, and protein quality control mechanisms. This review summarizes the currently available data on the epidemiology, clinical phenotypes, myopathology, and genetics of desminopathies. In addition, this work provides an overview on the expression, filament formation processes, biomechanical properties, post-translational modifications, interaction partners, subcellular localization, and functions of wild-type and mutant desmin as well as desmin-related cell and animal models.

## General introduction

Desminopathies (synonyms: desmin-related myopathy, desmin myopathy, desmin storage myopathy, and others [166]) belong to the clinically and genetically heterogeneous group of myofibrillar myopathies (MFM), which are morphologically characterized by the presence of desmin-positive protein aggregates and degenerative changes of the myofibrillar apparatus [160, 164]. Since the first description of desmin and αB-crystallin mutations causing MFM in 1998 [58, 187], an increasing number of MFM disease genes coding for sarcomeric and extra-sarcomeric proteins, i.e., BAG-3, FHL1, filamin-C, myotilin, plectin, titin, and ZASP, have been identified [126, 138, 160, 164]. In addition, protein aggregate myopathies due to mutations in the DNAJB6 and VCP genes share part of their morphological features with MFM [84, 151]. The precise molecular pathways leading from an individual MFM gene defect to a shared myopathological disease manifestation are still unclear.

Desmin is a member of the intermediate filament (IF) protein gene family, which comprises 70 members [78]. Up to now, the IF gene family represents one of the most highly mutated groups of related genes in the human genome, accounting for at least 94 different disease entities [132, 176]. IF proteins are expressed in a tissue- and development-specific manner, e.g., keratins in epithelial tissues, GFAP in astrocytes, and neurofilament proteins in neurons. As a particular unique property, the IF gene family harbors three genes that code for proteins localizing to the nuclear envelope within the cell nucleus, i.e., lamin A and its smaller splice form lamin C, lamin B1, and lamin B2. According to the degree of sequence identity, IF proteins have been grouped into six sequence homology classes (SHC): acidic keratins (SHC I); basic keratins (SHC II); desmin, vimentin and other mesenchymal IF proteins such as GFAP (SHC III); neurofilament proteins (SHC IV); lamins (SHC V); and an orphan group harboring the lens-specific IF proteins phakinin and filensin [72]. Human IF-related diseases range from skin blistering (keratins), Alexander’s disease (GFAP), Charcot-Marie-Tooth disease (neurofilament proteins), and Hutchinson-Gilbert progeria (lamin A/C) to cataracts (phakinin). Notably, and subject of this review, mutations of desmin, the major class III IF protein in striated and smooth muscle cells, causes progressive myopathy, cardiomyopathy, cardiac conduction defects, and arrhythmias [160, 183]. Information on the expression, localization, interaction, and function of IFs in general and desmin in particular is a prerequisite for the understanding of human desminopathies. In the present article we will summarize the essential data on desmin and desminopathies derived from numerous clinical and molecular studies.

## Clinical phenotypes

### Epidemiology

Since detailed epidemiological studies on MFM are not available, the incidence and prevalence of desmin myopathy and/or cardiomyopathy are currently unclear. Interpretation of the thus far published data allows the assumption that desminopathies fulfill the definition of a rare disease with no more than 5 affected individuals in 10,000. In a study on the prevalence of desmin mutations in a cohort of 116 families and 309 additional patients with pure dilatative cardiomyopathy, desmin mutations accounted for up to 2 % of disease manifestation [178]. Desmin mutations also seem to be one of the more frequently encountered gene defects in the MFM group. In a cohort of 53 patients from 35 Spanish MFM families, myotilin mutations were the predominant cause affecting 18 families followed by desmin mutations in 11 families [130]. In earlier studies reporting on the Mayo MFM cohort of 63 patients, 6 carried myotilin and 4 carried desmin mutations [165, 166]. Desminopathies have been reported in diverse ethnic groups and affect both female and male patients. Gender effects have been reported in two studies, in which male heterozygous mutation carriers were more prone to cardiac disease manifestations [5, 183]. The disease manifestation is highly variable with an age of onset ranging from the 1st to the 8th decade of life. In rare recessive forms the disease manifests in the 1st decade of life [31, 58, 121, 140]. In the more frequently encountered familial and sporadic cases due to heterozygous desmin mutations, a disease onset ranging from the 2nd to the 4th decade of life has been reported in the majority of patients [160, 183].

### Skeletal muscle disease

Initially, desminopathies have been associated with a progressive distal myopathy phenotype starting in the lower legs. However, subsequent studies reported the association between desmin mutations and limb girdle, scapuloperoneal, and generalized myopathy phenotypes [9, 35, 191]. A meta-analysis based on the interpretation of published data from 159 patients carrying 40 different heterozygous desmin mutations provided highly valuable insights into this complex issue [183]. Signs of combined distal and proximal muscular weakness were found in two thirds (67 %; 71/106) of mutation carriers, whereas true distal and proximal myopathy phenotypes were only present in 27 % (29/106) and 6 % (6/106), respectively. In this study, 74 % (110/148) of carriers had signs of skeletal muscle disease. Isolated skeletal muscle disease was reported in 22 % (31/141) of carriers. A combination of signs of skeletal muscle and cardiac pathology was found in 49 % (67/137).

Creatine kinase (CK) levels in desmin mutation carriers are of limited diagnostic value; 57 % (62/109) of mutation carriers had elevated CK levels (91 % displayed a ≤4-fold increase). Remarkably, 30 % (25/83) of patients with manifest skeletal muscle disease were reported to have normal CK levels [183]. Needle electromyography (EMG) typically reveals a myopathic pattern with short duration, polyphasic, and low amplitude motor unit potentials. In addition, positive sharp waves, fibrillation potentials, and pseudomyotonic/myotonic discharges have frequently been documented in desminopathy patients [160]. Sensory and motor nerve conduction studies usually give normal results [59]. Muscle MRI studies pointed out that signal alterations in the gluteus maximus, semitendinosus, sartorius, gracilis, and peroneal muscles are early and predominant signs of desminopathies [48, 156].

### Cardiac disease

Cardiac disease manifestations in desminopathies, which may precede, coincide with, or succeed skeletal muscle weakness, comprise true cardiomyopathy as well as various forms of cardiac conduction defects (CCD) and arrhythmias [92, 173, 178, 183, 185]. The above-cited meta-analysis revealed the presence of cardiological signs in 74 % (105/141) of desmin mutation carriers [183]. Isolated cardiological signs were reported in 22 % (34/152) of carriers. Out of 67 patients with verified cardiomyopathy (49 %; 67/138), 23 were classified as dilatative, 18 as unspecified, 16 as restrictive, 8 as hypertrophic, and 2 as arrhythmogenic right ventricular cardiomyopathy. Desmin mutations frequently lead to clinically symptomatic and asymptomatic ECG abnormalities (62 %; 83/133). In this group, CCD seems far more frequent (39 %; 52/133) than isolated arrhythmias (5 %; 6/133). A combination of both was found in 19 % (25/133) of carriers. A more detailed characterization of 77 patients with CCD revealed that atrioventricular block (47) and right bundle branch block (14) were the most prevalent manifestations. In 31 patients with arrhythmias, atrial fibrillation (9), ventricular premature beats (8), and ventricular tachycardia (7) were the most frequently detected ECG abnormalities. Thus, a basic electrophysiological workup should include a 12-lead surface ECG and a 24-h Holter ECG. The cardiac function is routinely assessed by transthoracic echocardiography. However, cardiac MRI has been proposed as a more sensitive diagnostic tool to detect focal cardiac pathology in early and clinically asymptomatic stages of desminopathy [173].

### Pulmonary and miscellaneous disease manifestations

Patients with desmin mutations are at risk to develop respiratory problems. In the desminopathy meta-analysis 26 % (29/110) of carriers were reported to suffer from respiratory insufficiency [183]. Thus, blood gas analysis and spirometry are mandatory in patients with desminopathies. Cataracts, swallowing difficulties, intestinal pseudo-obstruction, and repetitive episodes of diarrhea and constipation have been reported as miscellaneous or putative disease-related symptoms [6, 58, 129, 183].

### Disease progression and mortality

Desmin myopathies and cardiomyopathies are characterized by a progressive course and may change their initial clinical presentation. In a case report, the transformation of an initially hypertrophic cardiomyopathy into a restrictive and finally dilated cardiomyopathy has been documented [65]. A further study reported on a 10-year follow-up of 28 patients from 19 families with desmin mutations [190]. In 11 patients, primary skeletal muscle involvement was followed by cardiac disease after 6.3 ± 3.5 years, whereas in 16 patients with a primary cardiac disease manifestation skeletal muscle problems occurred after 6.8 ± 4.1 years. Out of the latter group, six patients presented with major cardiac complications. A progression from mild conduction defects to high degree conduction blocks requiring permanent pacing was observed in 8 out of 19 patients. In this cohort of 28 patients, 5 died at a mean age of 58.0 ± 6.5 years, accounting for a mortality rate of 17.8 % [190]. In the desminopathy meta-analysis, however, a mortality rate of 26 % (27/104) of desmin mutation carriers with a mean age 49 ± 9.3 years was reported [183]. Documented causes of death in both studies were sudden cardiac death, heart failure, respiratory insufficiency, chest infection, and iatrogenic complications of cardiac treatment. Both studies conclusively underline that the cardiac disease manifestation is the major cause of premature death in desminopathies.

## Muscle biopsy findings

### Light microscopy

**Fig. 1:**
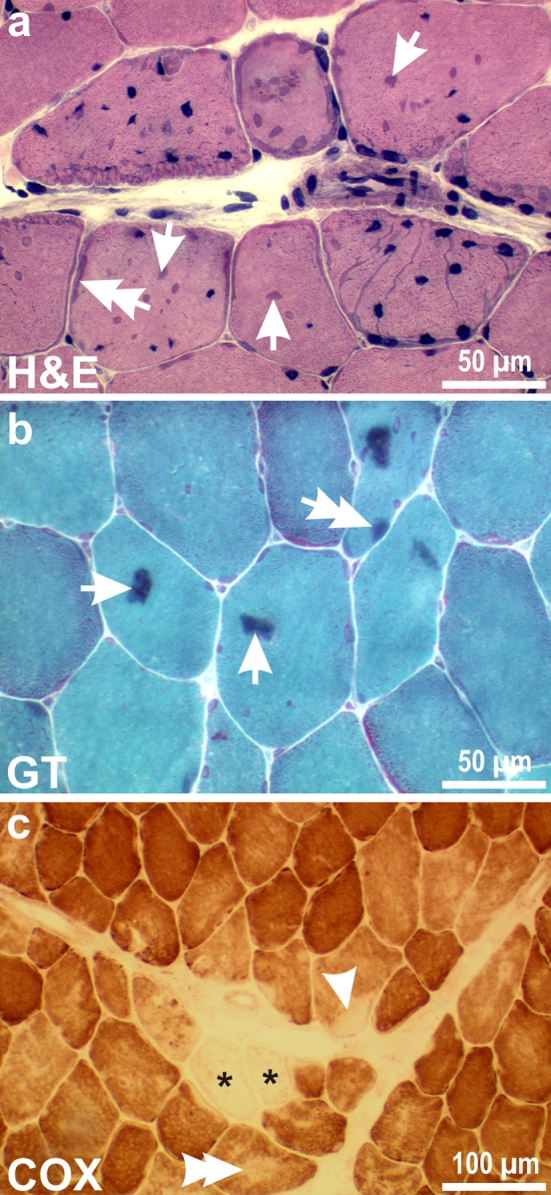
Protein aggregation pathology and mitochondrial abnormalities in desminopathies. *Arrows* and *double arrows* in **a** and **b** denote pathological protein aggregates in the sarcoplasm and in the subsarcolemmal region, respectively. *Arrowhead* and *double arrowhead* in **c** highlight a rubbed-out lesion and a core-like lesion, respectively

Diagnostic skeletal muscle biopsies from patients with desminopathies usually show mild to severe signs of a degenerative myopathy with rounding of muscle fibers, fiber splitting, internalization of myonuclei, and increased connective and fat tissue. Pathological protein aggregates, the hallmark of MFM, generally emerge as subsarcolemmal and/or sarcoplasmic inclusions. In addition, sarcoplasmic bodies as well as rimmed and non-rimmed vacuoles may be present. The typical pathology of MFM is best visualized in H&E and modified Gomori trichrome (GT) stains (Fig. 1a, b). Enzymatic stains (NADH-TR, SDH, and COX) may show further characteristic oxidative enzyme and mitochondrial abnormalities comprising rubbed-out fibers and core-like lesions (Fig. 1c). The stage of disease progression in individual muscles often mirrors the severity of the observed myopathological changes. However, one should keep in mind that the myopathological picture of desminopathies is highly variable. The myopathological findings in genetically proven desminopathies range from no overt pathology over subtle myopathic changes with or without pathological protein aggregates to the picture of a vacuolar myopathy [35, 160, 161].

### Immunodetection

**Fig. 2:**
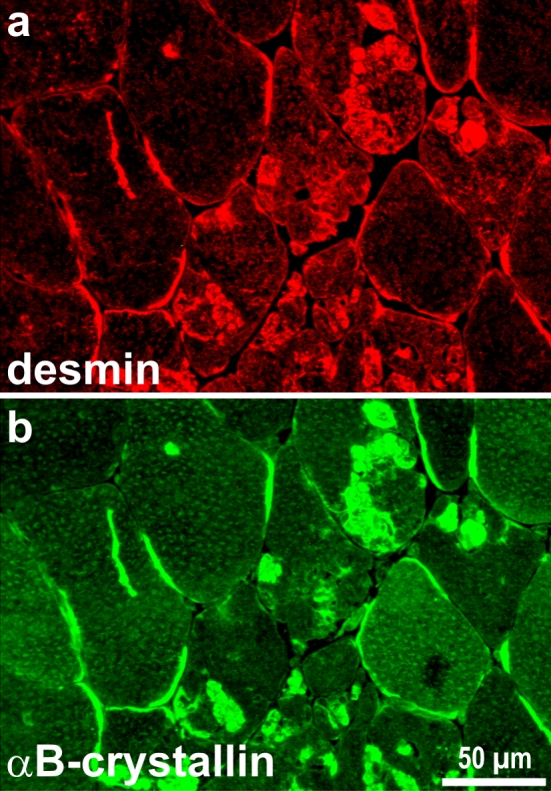
Indirect immunofluorescence labeling of desmin and αB-crystallin in a desminopathy. Note the presence of sarcoplasmic and subsarcolemmal pathological protein aggregates

Desmin immunostaining is mandatory to depict desmin-positive pathological protein aggregates in the subsarcolemmal and/or sarcoplasmic region. In addition to the characteristic immunoreactivity to desmin (Fig. 2a), the pathological protein aggregates are positively stained by a wide variety of antibodies directed against cytoskeletal proteins (e.g., dystrophin, F-actin, filamin C, myotilin, plectin, synemin), heat shock proteins (e.g., αB-crystallin, Hsp27), ubiquitin, Alzheimer-related proteins (e.g., β-APP), and cyclin-dependent kinases (e.g., CDK2, p21). Out of this long list, stains for αB-crystallin (Fig. 2b), filamin-C, and myotilin are sensitive diagnostic tools to depict pathological protein aggregation in desminopathies and other forms of MFM [161, 166].

### Electron microscopy

**Fig. 3:**
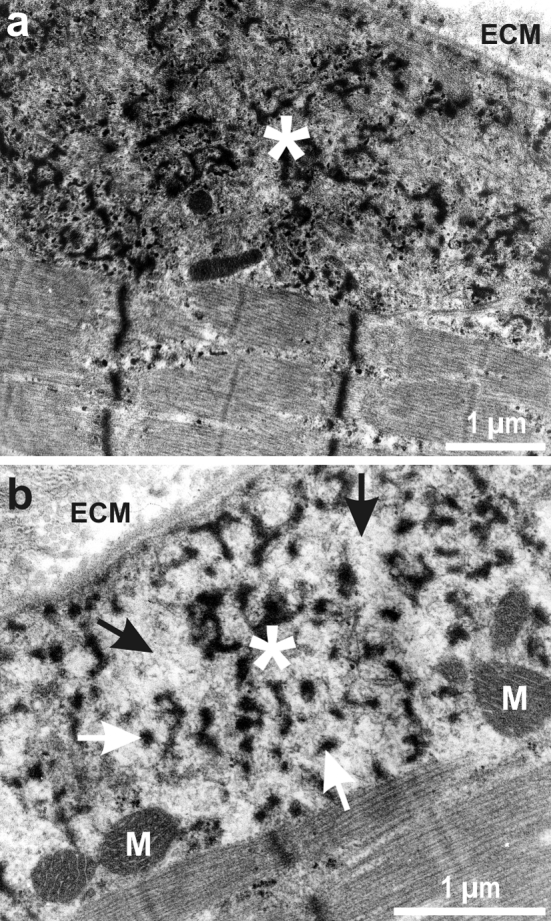
Electron microscopy findings in desminopathies. *Asterisks* denote the presence of granulofilamentous material in the subsarcolemmal region. *White arrows* depict electron-dense granular deposits; *black arrows* highlight filamentous material. *ECM* extracellular matrix, *M* mitochondrion

Granulofilamentous material in the subsarcolemmal region and/or between neighboring myofibrils is the classical ultrastructural hallmark of desminopathies (Fig. 3). However, granulofilamentous material is not specific for this disease entity as it has been described in other forms of MFM, in particular in αB crystallinopathies. Other features of pathological protein aggregation are cytoplasmic bodies and autophagic vacuoles. These changes are found in conjunction with signs of myofibrillar degeneration comprising Z-disc alterations (streaming, irregularities, loss, and rods), myofibrillar remnants, and core and core-like lesions. Typical signs of concomitant mitochondrial pathology are areas with accumulation or depletion of mitochondria with normal or abnormal morphology.

## Cardiac pathology

Desmin-positive protein aggregates as well as granulofilamentous and electron-dense amorphous materials are also the morphological hallmarks of desmin cardiomyopathies. Pathological aggregates have been demonstrated in subsarcolemmal, intermyofibrillar, and perinuclear regions. Further reported pathological findings were myocyte hypertrophy, disarray of the myocytes, mis-shaped myonuclei, cytoplasmic vacuolar degeneration, focally lysed myofibrils, various degrees of diffuse interstitial fibrosis, and mitochondrial abnormalities [3, 4, 20]. With respect to CDC and arrhythmias, pathological changes were noted in the cardiac conduction system of two autopsy cases. In one, calcifications at the bundle of His and calcium deposits at the left and right bundle branches were noted [199], whereas in the other extensive fibrosis in the terminal portion of the branching bundle and the initial segments of the left and right bundles were described [20]. The shape of intercalated discs has been reported to be abnormal with convoluted, elongated, and zigzag patterns [185]. Furthermore, decreased immunoreactivities of the desmosomal proteins desmoplakin and plakophilin-2 and the ventricular gap junctional protein connexin-43 have been described [133]. Remarkably, smooth muscle cells of cardiac blood vessels seem not to contain desmin-positive aggregates [4]. As in skeletal muscle biopsy, the extent of typical pathology is highly variable in cardiac specimens from desminopathies.

## Differential diagnosis

The differential diagnosis of desminopathies is complex and depends on the initial clinical disease presentation. Patients with a highly indicative phenotype of desminopathy (combined skeletal muscle and cardiac symptoms, no extra-muscular signs, autosomal dominant inheritance, disease onset between the 2nd and 4th decade of life) represent only a minority. In cases with isolated cardiac problems, a broad spectrum of acquired and hereditary conditions has to be taken into consideration. In cases of progressive skeletal muscle weakness without cardiac involvement, the differential diagnosis ranges from primary distal myopathies, limb girdle muscular dystrophies, and scapuloperoneal syndromes to generalized myopathies. A diagnostic muscle biopsy in these cases often provides the first clue to the diagnosis of MFM. Careful interpretation of the clinical data, including sex, age of onset, mode of inheritance, and presence or absence of extramuscular signs such as cataracts (αB-crystallinopathy), early respiratory failure (titinopathy), blistering skin (plectinopathy), and rigid spine and scoliosis (FHL1opathy, BAG-3opathy) is essential to differentiate specific MFM subtypes. In MFM patients with a disease onset beyond the 4th decade of life, mutations in genes coding for myotilin, ZASP, and filamin-C should initially be considered [160, 164].

## Genetics of desminopathies

### Human desmin gene

The human desmin gene (*DES*) on chromosome 2q35 is a single copy gene that spans over a length of approximately 8.3 kb and comprises nine exons coding for a 470-amino acid protein with a molecular weight of 53.5 kDa [106]. *DES* belongs to a gene cluster further comprising *APEG1* (synonym *SPEG* or striated muscle preferentially expressed protein kinase) and *CHPF*. This gene cluster is most likely regulated by the *DES* 5′ locus control region (LCR), which has been identified 9–18 kb upstream of *DES* [177]. The currently available information on the epigenetic regulation of *DES* is still limited. The *DES* promoter contains a CpG-island also covering the *DES* transcription start site and exon 1. It has been described that the *DES* promoter is non-methylated regardless of its expression status. The expression of desmin in muscle cells seems to be activated by acetylation of histones H3 and H4 as well as methylation of histone H3 at lysine residue 4 (H3K4me2 and me3) around the transcription start site of *DES*. In non-muscle cells, the *DES* promoter is silenced by methylation of histone H3 lysine residue 27 (H3K27me3) [108].

### Desmin mutation spectrum

**Fig. 4:**
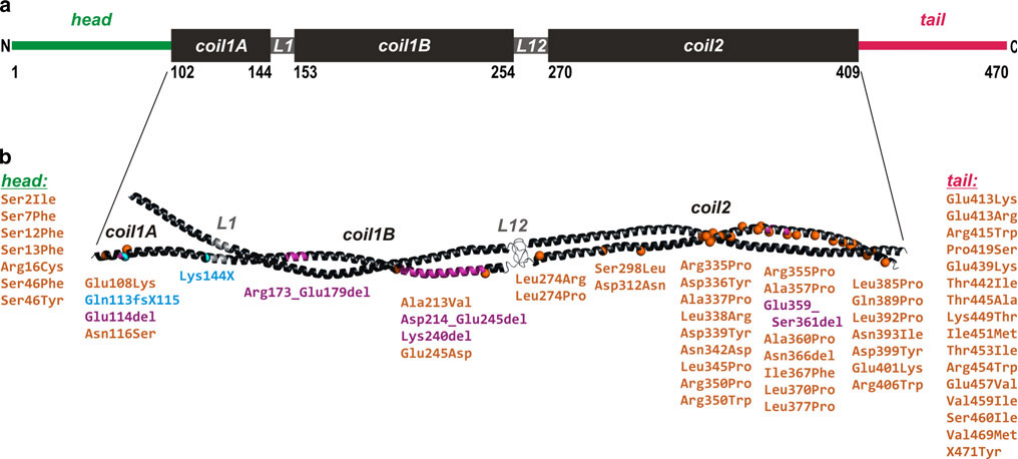
Structural organization of the human desmin molecule and molecular model of its dimeric rod domain. **a** *The black boxes* represent α-helical segments designated “coil.” Segments of unknown structure connecting coil 1A and coil 1B as well as coil 1B and coil 2 are termed linkers L1 and L12, respectively. Non-structured amino- (“head”) and carboxy- (“tail”) terminal domains are depicted as colored bars. *Numbers* indicate the amino acid position of the domain borders. **b** The molecular model of the dimeric desmin coiled coil domain is based on its high structural homology to the corresponding vimentin domain [33]. The three α-helical segments (coil 1A, coil 1B and coil 2) are shown as *black ribbons*. The linkers L1 and L12 are in *grey*. The first segment (coil 1A) has only a weak tendency to form coiled coils [33]. Locations of pathogenic mutations are mapped in *orange* (missense mutations), *purple* (deletions), and *cyan* (truncations). For clarity, mutation sites in only one chain of the dimer are marked. The mutations within the unstructured head and tail domains are also listed; see also Table 1

Disease-causing mutations spread over the entire *DES* gene. They significantly cluster in exon 6, which encodes the C-terminal half of the coil 2 domain (Fig. 4). Mutations in this domain have primarily been associated with a skeletal muscle phenotype, whereas mutations residing in the head and tail domains of the desmin protein seem more frequently associated with a cardiac phenotype [183]. As of June 2012, 67 disease-causing mutations of the *DES* gene have been published. These mutations may lead to the expression of 61 different mutant forms of desmin (Table 1).

**Table 1:** Human desmin mutations, clinical phenotypes, myopathology, and related in vitro data

Row no.	Domain	Mutation	Mutation type	Mode of inheritance	Phenotype	Histology	References
1	Head	Ser2lle	Missense	AD	gM, cM, ri	sva	[167, 190]
2		Ser7Phe	Missense (Fig. 2 of reference)	AD	n.a.	n.a.	[55, 56]
3		Ser12Phe	Missense	AD	pM, dM, cM, ary	sva, atf, intN, rvac	[83]
4		Ser13Phe	Missense	AD	pM, dM, cM, ary	sva, atf, intN, rvac	[21, 139, 167, 184, 185]
5		Arg16Cys	Missense	AR	cM, ary	atf, intN, rvac	[4, 167]
6		Ser46Phe	Missense	AD	n.a.	sva	[167]
7		Ser46Tyr	Missense	AD	n.a.	sva	[167]
8	Coil 1A	Glu108Lys	Missense	AD	cM, ary	n.a.	[178]
9		Gln113fsX115	Frame shift	SP	gM, cM, ary	sva, intN	[83]
10		Glu114del	Small in-frame deletion	AD	gM, cM, ary	sva, intN, ppa	[186]
11		Asn116Ser	Missense	SP	(gM), cM, ary	sva, atf	[92]
12		Lys144X	Frame shift	AD	gM, cM, ary, ri	n.a.	[190]
13	Coil 1B	Arg173_Glu179del	Small in-frame deletion	AR	gM, cM, ri, ary	atf, intN, ppa	
14		Ala213Val	Missense, putative polymorphism	AD, SP	pM, dM, cM, ary	Necrosis, ppa	[57, 62, 95]
15		Asp214_Glu245del	Splice site mutations leading to loss of exon 3: c.640-2A>T, c.640-1 G>A, c.735G>C, c.735G>T, c.735 + 1G>A, c.735 + 3A>G	AD, SP	gM, pM, cM, 4par, ary, ri	sva, rvac, ppa	[35, 42, 59, 64, 135, 190]
16		Lys240del	Small in-frame deletion, corrected from p.Lys239fsX242 (*)	AD	gM pM, ary	sva, ppa	[158, 159]
17		Glu245Asp	Missense	AD	gM 4par, cM, ary, (ri)	sva, atf, intN, rvac	[35, 66, 173, 189]
18	Coil 2	Leu274Arg	Missense	AD	gM cM, ary	sva, intN	[83]
19		Leu274Pro	Missense	AD	gM cM, ary	sva, intN	[83]
20		Ser298Leu	Missense	AD	cM ary	n.a.	[178]
21		Asp312Asn	Missense	AD	cM	n.a.	[178]
22		Arg335Pro	Missense	AD	gM ary	sva, intN	[83]
23		Asp336Tyr	Missense	AD	gM cM, ary	n.a.	[190]
24		Ala337Pro	Missense	AD	gM pM, dM, cM, ary, ri	atf, intN, ppa	[42, 58, 62, 199]
25		Leu338Arg	Missense	AD	gM pM, dM, ri	ppa	[62, 190]
26		Asp339Tyr	Missense	SP	dM ri	n.a.	[173]
27		Asn342Asp	Missense	AD, SP	gM pM, dM, cM, ri, ary	atf, intN, ppa	[41, 42, 133, 184, 190]
28		Leu345Pro	Missense	AD	pM dM, cM, ary	sva, atf, intN, rvac	[169, 190]
29		Arg350Pro	Missense	AD	gM pM, dM, spM, cM, ary	sva, atf, intN	[12, 173]
30		Arg350Trp	Missense	AD	cM	n.a.	[178]
31		Arg355Pro	Missense	AD	gM pM,dM,cM	sva, atf, intN	[47, 190]
32		Ala357Pro	Missense	AD	gM pM, dM, spM, ri	atf, ppa	[39]
33		Glu359_Ser361del	Small in-frame deletion	AD	gM pM,dM	atf, ppa	[89]
34		Ala360Pro	Missense, compound heterozygote (Asn393lle)	AR	gM pM, dM, cM, ary, ri	atf, intN, ppa	[42, 58, 62]
35		Asn366del	Small in-frame deletion	AD	gM pM, dM, cM, ary, ri	ppa	[89, 129]
36		Ne367Phe	Missense	AD	gM pM, dM, cM, ary, ri	sva, intN, rvac, ppa	[128]
37		Leu370Pro	Missense	AD	gM pM, dM, cM, ary, ri	sva, atf, intN, rvac, ppa	[5, 39]
38		Leu377Pro	Missense	SP	gM cM, ri	n.a.	[173]
39		Leu385Pro	Missense	SP	gM cM, ary, ri	sva	[174]
40		Gln389Pro	Missense	SP	pM dM, cM, ary	sva, ppa	[62]
41		Leu392Pro	Missense	AD	gM pM, dM, cM, ary, ri	sva, intN, rvac, ppa	[128]
42		Asn393lle	Missense, compound heterozygote (Ala360Pro)	AR	gM cM, ary, ri	atf, intN, ppa	[42, 58, 62]
43		Asp399Tyr	Missense	AD	pM dM, cM, ri, ary	atf, ppa	[62]
44		Glu401Lys	Missense	SP	gM pM, dM, cM, ary, ri	atf, ppa, rvac	[62]
45		Arg406Trp	Missense	AD, SP	gM pM, dM, cM, ary, ri	atf, intN	[4, 40, 42, 129, 136, 190]
46	Tail	Glu413Lys	Missense	AD	gM pM, dM, cM, ary	sva, atf, intN, ppa	[10, 144]
47		Glu413Arg	Missense	AD	gM cM, ary	n.a.	[190]
48		Arg415Trp	Missense (Fig. 2 of reference)	AD	n.a	n.a.	[55, 56]
49		Pro419Ser	Missense	AD	gM pM, dM,cM, ary	sva, intN, rvac, ppa	[69, 128, 190]
50		Glu439Lys	Missense	AD	gM cM, ary	n.a.	[190]
51		Thr442lle	Missense	AD, SP	gM pM, dM, cM, ary	atf, intN, rvac	[10, 14, 175, 190]
52		Thr445Ala	Missense	SP	gM ri	atf, intN, rvac	[83]
53		Lys449Thr	Missense	AD, SP	n.a	n.a.	[10, 14]
54		lle451Met	Missense	AD, SP	gM pM, dM, cM, ri	atf, intN, rvac, ppa	[14, 41, 42, 102, 118]
55		Thr453lle	Missense	AD, SP	cM ary	ppa	[4]
56		Arg454Trp	Missense	AD, SP	gM pM, dM, cM, ary, ri	atf, ppa	[10, 133, 190]
57		Glu457Val	Missense	AD	gM cM, ary	sva, atf, intN	[83]
58		Val459lle	Missense	AD	cM ary	n.a.	[190]
59		Ser460lle	Missense	AD	pM dM, cM, ary	sva, atf, rvac	[10, 14]
60		Val469Met	Missense	AD	pM dM,cM	n.a.	[10, 14]
61		X471Tyr	Missense/loss of stop codon	AD	pM dM, ary	n.a.	[190]

Row no.	Immunostains	Ultrastructure	Assembly of Mut	Assembly mix Mut/WT	Tranfection into IF negative cell	Tranfection into IF positive cell	References
1	n.a.	n.a.	FNF, (Irw)	FNF	FNF (SW13), FNF (Vim^K0^ MEF)	FNF (HL-1)	[167, 190]
2	n.a.	n.a.	n.a.	n.a.	n.a.	n.a.	[55, 56]
3	des, abc, dys	GFM	n.a.	n.a.	Agg (SW13)	Agg (C2C12)	[83]
4	des	Microgranular densities	Agg	FNF	FNF/Agg (MCF7), SFF (SW13), FNF/Agg (Vim^K0^ MEF)	FNF/Agg (BHK21), Agg (HL-1)	[21, 139, 167, 184, 185]
5	des	Dense material	Agg	FNF	FNF (SW13), FNF/Agg (Vim^K0^ MEF)	Agg(HL-1)	[4, 167]
6	n.a.	n.a.	FNF, (Irw)	FNF	SFF (SW13), FNF (Vim^K0^ MEF)	FNF (HL-1)	[167]
7	n.a.	n.a.	FNF, (Irw)	FNF	SFF (SW13), FNF (Vim^K0^ MEF)	FNF (HL-1)	[167]
8	n.a.	n.a.	n.a.	n.a.	FNF (SW13)	FNF (hcasmc, nrcm)	[178]
9	des, abc, dys, hyalin	GFM	n.a.	n.a.	Agg (SW13)	Agg (C2C12)	[83]
10	des	GFM	n.a.	n.a.	Agg (SW13), Agg(3T3)	Agg (C2C12)	[186]
11	des, myotilin	GFM	Agg	FNF, Agg	Agg (SW13)	n.a.	[92]
12	n.a.	n.a.	n.a.	n.a.	n.a.	n.a.	[190]
13	des	GFM	SFF	SFF	n.a.	Agg (MCF7)	[57, 121, 140]
14	n.a.	n.a.	Agg		FNF (SW13), Agg (BMGE + H)	FNF(C2C12)	[57, 62, 95]
15	des	GFM	n.a.	n.a.	n.a.	n.a.	[35, 42, 59, 64, 135, 190]
16	des, syn, pic, ubi	GFM	n.a.	n.a.	Agg (SW13)	Agg (BHK21)	[158, 159]
17	des	GFM	n.a.	n.a.	n.a.	n.a.	[35, 66, 173, 189]
18	des, abc, dys	GFM	n.a.	n.a.	Agg (SW13)	Agg (C2C12)	[83]
19	des, abc, dys	GFM	n.a.	n.a.	Agg (SW13)	Agg (C2C12)	[83]
20	n.a.	n.a.	n.a.	n.a.	Agg (SW13)	Agg (hcasmc, nrcm)	[178]
21	n.a.	n.a.	n.a.	n.a.	Agg (SW13)	Agg (hcasmc, nrcm)	[178]
22	des	GFM	n.a.	n.a.	Agg (SW13)	Agg (C2C12)	[83]
23	n.a.	n.a.	n.a.	n.a.	n.a.	n.a.	[190]
24	des	GFM	n.a.	n.a.	Agg (SW13)	Agg (C2C12)	[42, 58, 62, 199]
25	n.a.	n.a.	n.a.	n.a.	Agg (SW13)	Agg (C2C12)	[62, 190]
26	n.a.	n.a.	n.a.	n.a.	n.a.	n.a.	[173]
27	des	GFM	n.a.	n.a.	Agg (SW13)	n.a.	[41, 42, 133, 184, 190]
28	des, vim, nestin	GFM	n.a.	n.a.	Agg (SW13)	Agg (HeLa)	[169, 190]
29	des, abc, pic	GFM	ULF	Agg	Agg (MCF7, SW13, BMGE + H)	Agg (3T3-L1)	[12, 173]
30	n.a.	n.a.	n.a.	n.a.	Agg (SW13)	Agg (hcasmc, nrcm)	[178]
31	des, abc, dys, hyalin	GFM	n.a.	n.a.	n.a.	n.a.	[47, 190]
32	des	GFM	n.a.	n.a.	Agg (SW13)	Agg (BHK21)	[39]
33	abc	GFM	n.a.	n.a.	Agg(SW13)	Agg (BHK21)	[89]
34	des	GFM	n.a.	n.a.	Agg (SW13)	FNF (C2C12)	[42, 58, 62]
35	des	GFM	n.a.	n.a.	Agg (SW13)	Agg (BHK21)	[89, 129]
36	des, abc, fine, dys	GFM	n.a.	n.a.	n.a.	n.a.	[128]
37	des	Disrupted myofibrils, GFM	n.a.	n.a.	Agg (SW13)	Agg (BHK21)	[5, 39]
38	n.a.	n.a.	n.a.	n.a.	n.a.	n.a.	[173]
39	des	GFM	n.a.	n.a.	n.a.	Agg/cell death (COS-7, CHO)	[174]
40	des	GFM	Agg	Agg	Agg (SW13)	Agg (MCF7, C2.7)	[62]
41	des, abc, fine, dys	GFM	n.a.	n.a.	n.a.	n.a.	[128]
42	des	GFM	n.a.	n.a.	Agg (SW13)	Agg (C2C12)	[42, 58, 62]
43	n.a.	n.a.	n.a.	n.a.	Agg (SW13)	Agg (C2C12)	[62]
44	n.a.	n.a.	n.a.	n.a.	Agg (SW13)	Agg (C2C12)	[62]
45	des	GFM	n.a.	n.a.	Agg (SW13)	n.a,	[4, 40, 42, 129, 136, 190]
46	des	GFM	ULF	FNF, SFF	Agg (SW13)	Agg (C2C12)	[10, 144]
47	n.a.	n.a.	n.a.	n.a.	n.a.	n.a.	[190]
48	n.a.	n.a.	n.a.	n.a.	n.a.	n.a.	[55, 56]
49	des, abc, fine, dys	GFM	n.a.	n.a.	n.a.	n.a.	[69, 128, 190]
50	n.a.	n.a.	n.a.	n.a.	n.a.	n.a.	[190]
51	des	GFM	FNF	FNF	FNF (SW13), FNF (Vim^K0^ MEF)	FNF (C2C12, HL-1)	[10, 14, 175, 190]
52	des, abc, dys, hyalin	GFM	n.a.	n.a.	Agg (SW13)	Agg (C2C12)	[83]
53	n.a.	FNF	FNF	FNF	FNF (Vim^K0^ MEF)	FNF (HL-1)	[10, 14]
54	des	FNF	FNF	FNF	FNF (SW13), FNF (Vim^K0^ MEF)	FNF (SW13, C2.7, MCF7, HL-1)	[14, 41, 42, 102, 118]
55	des	Dense material	n.a.	n.a.	n.a.	n.a.	[4]
56	des	GFM, NSA	SFF	FNF	Agg (SW13)	FNF (C2C12)	[10, 133, 190]
57	des, abc, dys, hyalin	GFM	n.a.	n.a.	Abnormal FNF (SW13)	Aberrant FNF (C2C12)	[83]
58	n.a.	n.a.	n.a.	n.a.	FNF/Agg (SW13)	Agg (hcasmc, nrcm)	[190]
59	des	GFM	FNF	FNF	Agg (SW13), FNF (Vim^K0^ MEF)	FNF (C2C12, HL-1)	[10, 14]
60	n.a.	FNF	FNF	FNF	FNF (SW13), FNF (Vim^K0^ MEF)	FNF (C2C12, HL-1)	[10, 14]
61	n.a.	n.a.	n.a.	n.a.	n.a.	n.a.	[190]

As of June 2012, 67 disease causing mutations of the *DES* gene have been published, which may give rise to the expression of 61 different mutant forms of the desmin protein. In addition, two French patients with a virtually complete lack of desmin protein expression due to a homozygous 22-bp deletion in exon 6 (mutation not further specified, therefore not listed in Table 1) have been described [31]. GenBank entry NM_001927.3 was used as desmin reference sequence; nucleotide numbering refers to the cDNA sequence with +1 corresponding to the A of the ATG start codon. Note of caution (*): The p.Lys239fsX242 insertion mutation, which subsequently had been corrected into p.Lys240del, erroneously has been cited as “ins245” [137] or “Glu245X” in few later publications [27, 178]. Cell types in brackets in the columns “Transfection into” are described in the main text

Abbreviations in alphabetical order: *4par* tetraparesis, *abc* aB-crystallin, *AD* autosomal dominant, *Agg* aggregates, *AR* autosomal recessive, *ary* cardiac arrhythmia, *atf* atrophic fibres, *cM* cardiac myopathy, *des* desmin, *dM* distal myopathy, *dys* dystrophin, *flnc* filamin-C, *FNF* filament/network formation, *GFM* granulofilamentous material, *gM* generalized myopathy, *intN* internalized nuclei, *Irw* irregular filament width, *n.a.* not available, *NSA* negative-stained assemblies, *plc* plectin, *pM* proximal myopathy, *ppa* pathological protein aggregates/inclusions/deposits, *ri* respiratory insufficiency, *rvac* rimmed vacuoles, *SFF* short filament formation, *SP* sporadic, *spM* scapuloperoneal myopathy, *sva* size variability, *syn* synemin, *ubi* ubiquitin, *ULF* unit length filaments/ULF-like blobs, *vim* vimentin

### Autosomal dominant inheritance

The vast majority of familial desminopathies follow an autosomal dominant mode of inheritance. The most frequent *DES* mutations are missense mutations leading to single amino acid substitutions. Splice site mutations causing the loss of exon 3 (p.Asp214_Glu245del), small in-frame deletions of one, three or seven codons, and frame shift mutations, which may lead to the expression of truncated desmin protein species, have been reported only in a small number of patients (Table 1). For the p.Arg350Pro mutation, the most frequently encountered pathogenic desmin missense mutation in Germany, a founder allele has been established [191].

### Autosomal recessive inheritance

Five families with an autosomal recessive mode of inheritance have been published thus far. In one family, the homozygous Arg16Cys missense mutation was observed [4]. In two families, a small in-frame deletion has been identified leading to p.Arg173_Glu179del [121, 140]. In another family, a 22 base pair deletion in exon 6 causing a premature stop codon associated with a virtually complete lack of desmin protein has been reported [31]. In the fifth family, the disease manifestation has been attributed to a compound heterozygote *DES* mutation (p.Ala360Pro/p.Asn393Ile [58]) (Table 1).

### Sporadic forms

In addition to these familial cases, an increasing number of sporadic desminopathies have been published. These sporadic disease manifestations are due to missense, splice site, or frame-shift mutations (Table 1).

## Desmin protein

### Protein structure and filament assembly

Human desmin is a 470-amino acid protein ([79, 153]; UniProtKB/Swiss-Prot database entry P17661) with a calculated molecular weight of 53.5 kDa. Like all IF proteins, it is fibrous in nature, exhibiting a tripartite structure with a central, mostly α-helical domain of conserved size, i.e., ~45 nm. This so-called “rod” is flanked by non-α-helical amino-terminal (“head”) and carboxy-terminal (“tail”) domains of highly varying amino acid number [73] (Fig. 4a). The “rod” domain comprises two continuous α-helical segments, coil 1 and coil 2, which are connected by a “linker” (L12) of unknown structure. The current structural model of desmin is derived from recent structural analyses of the closely related class III IF protein vimentin [33] (Fig. 4b). Coil 1 consists of two α-helical subdomains, i.e., coil 1A and coil 1B. They are separated by linker L1, which could also accommodate α-helical conformation. While coil 1B forms a stable dimeric coiled coil, the coil 1A segment has only a weak coiled-coil forming capacity. In addition, the current model omits the previously used separation into coil 2A and 2B domains, but demonstrates it to be a continuous α-helix [123].

**Fig. 5:**
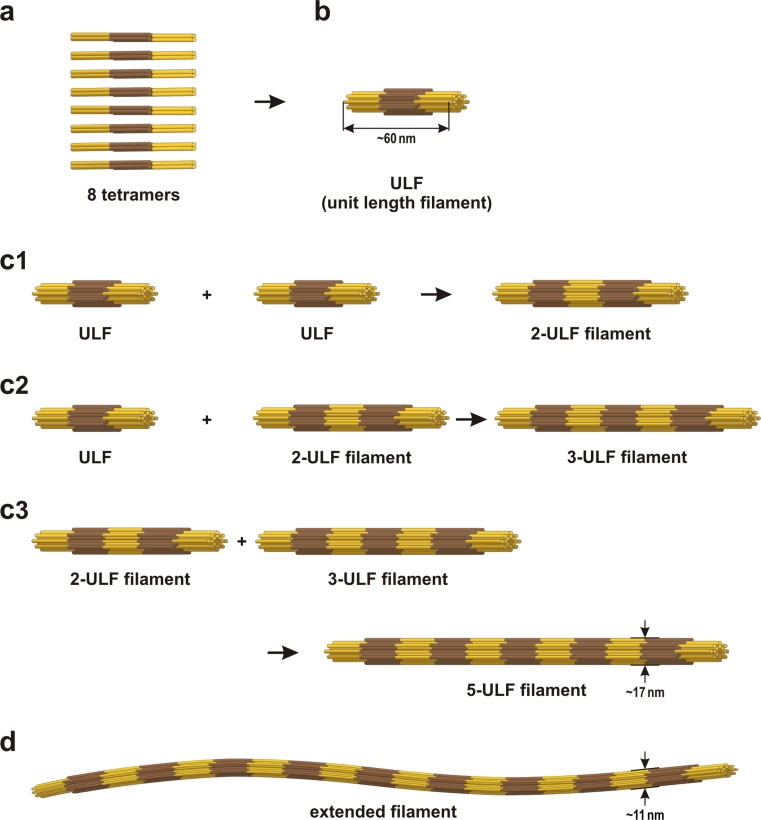
Schematic model of cytoplasmic IF assembly. **a** Two dimers associate in an anti-parallel, half-staggered fashion by overlap of the two coiled coils via their coil1, i.e., coil 1A and coil 1B (*brown segments*). **b** Upon initiation of assembly conditions the tetramers associate laterally into a unit-length filament (ULF). In vimentin, a ULF counts eight tetramers on the average [76]. **c** Individual ULFs may longitudinally anneal with another ULF (c1) or with a filament consisting of two annealed ULFs (c2); eventually, two short filaments may longitudinally anneal to a longer filament (c3). **d** Upon extended incubation, long filaments will spontaneously reduce their diameter by radial compaction to form a mature IF [76]. The figure is redrawn and modified after [79]

Desmin is a highly insoluble protein and hence it can be kept in solution as a monomer and purified only under highly denaturing conditions as provided by buffers containing 8 M urea. For assembly, it is usually first re-natured by dialysis into buffers of low salt such as 2 mM sodium phosphate (pH 7.5). The assembly process starts actually during the course of re-naturation, and two molecules dimerize by coiled-coil formation of the central α-helical rod domains of two desmin monomers in parallel orientation [77]. A so-called heptad repeat pattern, which is characteristic for a coiled-coil structure [26], drives the supercoiling of the two helices, yielding the dimeric, elongated structure. Two of these coiled-coil dimers further associate in a half-staggered, anti-parallel fashion to a tetramer of ~60 nm length [77, 107] (Fig. 5a). These tetramers are able to spontaneously assemble into highly ordered long filaments upon increase of the ionic strength to physiological values. The assembly process can be described to occur in three phases: (1) The lateral parallel assembly of tetramers yielding full-width, 60-nm-long filaments that have been termed unit-length filaments or ULFs [71, 77] (Fig. 5b). (2) The formation of extended intermediate filaments by serial longitudinal annealing of ULFs and further longitudinal annealing of short filaments [71] (Fig. 5c1,c2,c3,d). (3) After a few minutes of assembly, filaments undergo a final maturation step characterized by a radial compaction process [76, 79].

### Protein expression

Desmin is the most abundant IF protein in striated and smooth muscle cells. Immunoblotting after one-dimensional SDS-PAGE with desmin-specific antibodies shows a single band corresponding to an apparent molecular weight of 58 kDa. In addition to muscle, desmin expression has been described in a wide variety of normal and diseased cells, for example, pericytes [24], hepatic stellate cells [124], myoid stromal cells of placenta [170], Sertoli cells [148], decidual cells [67], injured glomerular podocytes [70], and mesotelioma cells [85].

Desmin is one of the earliest markers of muscle development [27], and this is true for all vertebrates including amphibian and fish, pointing to an important evolutionary conservation [75, 153]. Its expression precedes all other muscle-specific structural proteins and even—with the exception of myf-5—the expression of the myogenic transcription factors myoD, myogenin, and mrf4 (myf-6) [99, 103, 112]. During early muscle development desmin and vimentin are co-expressed. Vimentin is the most abundant IF protein of immature myoblasts. Upon further differentiation into mature muscle cells, desmin is strongly upregulated, while the expression of vimentin is completely ceased [43, 152].

### Post-translational modifications

Post-translational modifications do play an important role in regulating the functions of intermediate filaments. In vitro translation of chicken skeletal and smooth muscle desmin using a cell-free rabbit reticulocyte system resulted in predominantly non-phosphorylated desmin, suggesting a low amount of phosphorylated desmin under basal conditions [125]. On the other hand, desmin has been described as one of the major acceptors of ^32^P in differentiating chicken myotubes. Two-dimensional tryptic analysis of desmin revealed multiple sites of phosphorylation [52]. Another study showed that desmin is a substrate of the p21-activated kinase (PAK) mainly leading to phosphorylation of serine residues within the desmin “head” domain. PAK-mediated phosphorylation of desmin strongly inhibited its filament-forming ability [127]. Assembly is also inhibited by ADP ribosylation of desmin via an arginine-specific mono-ADP-ribosyltransferase of muscle, which primarily targets arginine 48 and to a lesser extent arginine 68 within the desmin “head” domain [201]. A subsequent study provided evidence that ADP-ribosylated desmin neither co-assembles with nor affects the filament formation of non-modified desmin. In contrast, the ADP-ribosylation of pre-formed desmin IFs resulted in their disassembly. Furthermore, this process is dependent on the phosphorylation of additional amino acid residues [198].

### Subcellular localization and functions

Desmin immunostaining of cross-sectioned striated muscle revealed sarcoplasmic and subsarcolemmal localizations. In longitudinal sections, desmin immunostains revealed a cross-striated pattern [162]. In addition, desmin is enriched at the level of myotendinous and neuromuscular junctions of skeletal muscle as well as of intercalated discs in cardiac muscle [30, 180]. Immunogold electron microscopy demonstrated the presence of desmin at costameres, filamentous structures spanning between myofibrils and the overlaying sacolemma, filamentous inter-Z-disc structures of neighboring myofibrils and mitochondria, and at the desmosomal plaque of intercalated discs, where desmin IFs bind to desmoplakin, a member of the plakin family of crossbridging proteins [90, 111, 146, 162].

**Fig. 6:**
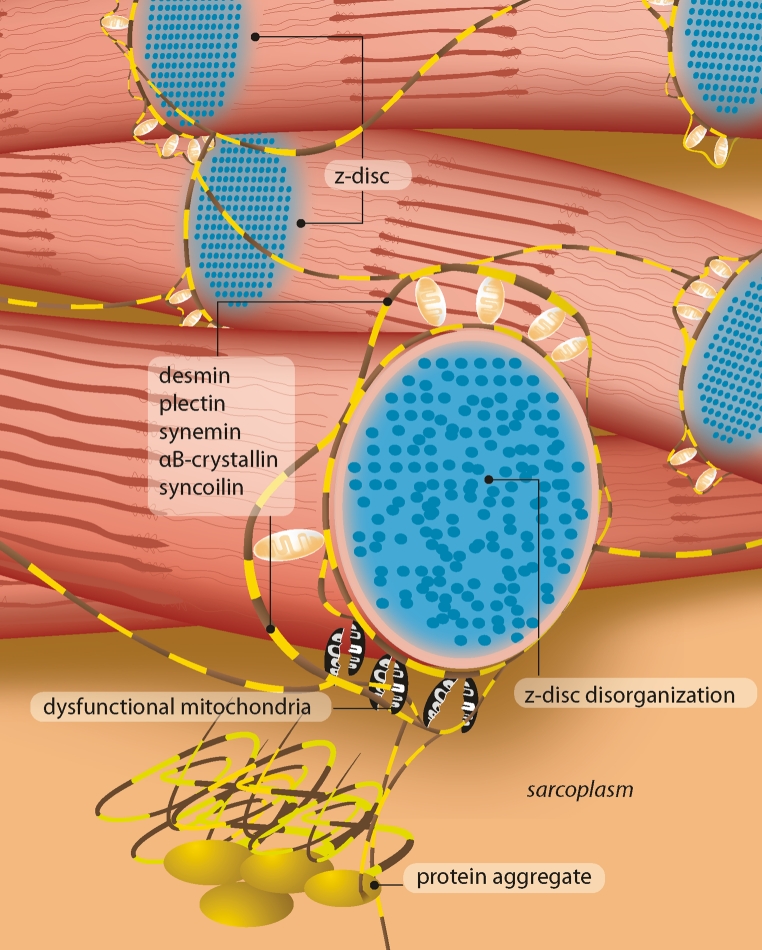
Schematic of the desmin IF network in relation to the myofibrillar apparatus. In desminopathies, mutant desmin leads to structural and functional changes of the extrasarcomeric cytoskeleton, pathological protein aggregation, mitochondrial abnormalities, and signs of myofibrillar degeneration

This three-dimensional filamentous extra-sarcomeric desmin cytoskeleton interlinks neighboring myofibrils (Fig. 6) and connects the myofibrillar apparatus with myonuclei, other cell organelles, and the extracellular matrix via the subsarcolemmal cytoskeleton [27, 160]. Beyond a sole mechanical integration, this complex interaction is thought to be the basis for a mechano-chemical signaling between various compartments. IFs in general and desmin in particular have also been hypothesized to directly bind to genomic DNA and to exert a role in transcription regulation and DNA organization [182]. Roles of desmin in the morphology and homeostasis of myonuclei, mitochondria, and lysosomes are discussed in the respective paragraphs on muscle pathology, desmin binding partners, and desmin mouse models. Biomechanical aspects of desmin at the level of single IFs, myoblasts, myofibers, and whole muscle are summarized in the respective paragraph on biomechanics.

### Molecular interaction partners

Desmin exerts its multiple functions through direct and indirect binding to various other cellular molecules. In the past 4 decades of desmin research, a growing number of desmin binding partners has been described (see also a previous review on desmin interactions [38]). The following description of desmin interactions is confined to binding partners that are present in muscle cells, i.e., IF proteins, IF-associated proteins, sarcomeric proteins, membrane-associated proteins, small heat shock proteins, apoptosis-related proteins, and nucleic acids.

#### Interactions with other IF proteins

During muscle development and maintenance, desmin has been reported to interact with five IF proteins, i.e., vimentin, nestin, synemin (also known as desmuslin), and syncoilin, which all are expressed in a spatiotemporal pattern. In addition, the nuclear B-type lamins have been claimed to be a direct binding partner of desmin [54, 111]. With the cytoplasmic IF-proteins the situation is much clearer: Desmin and vimentin are closely related class III IF proteins, whereas nestin, synemin, and syncoilin are more distantly related class IV IF proteins. Desmin, vimentin, and nestin are co-expressed and colocalized in myoblasts. Vimentin and nestin have each been demonstrated to assemble into “heteropolymeric” IFs with desmin [74, 79, 172]. While vimentin expression is downregulated, desmin and nestin are further expressed in later stages of myogenesis [168]. In contrast to desmin, nestin is primarily retained at myotendinous and neuromuscular junctions in mature skeletal muscle [28]. Moreover, synemin was found to co-purify and colocalize with desmin and vimentin [63]. However, synemin seems not to participate in the formation of mixed filaments, but binds to pre-formed desmin or vimentin IFs [18, 81, 150]. In mature skeletal muscle, synemin is localized at the periphery of Z-discs and at the sarcolemma ([119], and own observations). Another IF protein that directly interacts and colocalizes with desmin in mature skeletal muscle is syncoilin, which is localized at neuromuscular junctions, the sarcolemma, and Z-discs. Similar to synemin, syncoilin binds to IFs but does not participate in the formation of mixed filaments and therefore should be called an IF-associated protein. Syncoilin has been postulated to play a role in the cytoskeletal anchorage of the desmin IF [142].

#### Interactions with IF-associated proteins

Desmin directly interacts with multiple IF-associated proteins. The proper structural and functional organization of the desmin IF system highly depends on the interaction of desmin with several plectin isoforms. In skeletal muscle, the two plectin isoforms 1d and 1f link desmin IFs to Z-discs and costameres, respectively, whereas isoform 1b links desmin to mitochondria [93]. Plectin has been reported to directly bind to the coiled-coil rod domain of desmin via its fifth plakin repeat domain and part of the following linker domain [45]. During myodifferentiation desmin has been shown to co-purify and colocalize with paranemin—the chicken ortholog of mammalian nestin [147]—at the level of Z-discs in myotubes. In contrast to plectin but similar to nestin, paranemin is downregulated upon differentiation and absent in mature muscle cells [25]. Paranemin has been described to associate with desmin IFs—but not to form heteropolymers—leading to the formation of an extended desmin IF network [163]. A further direct desmin binding protein is myospryn (or cardiomyopathy-associated protein 5), a component of the biogenesis of lysosome-related organelles complex 1 (BLOC-1). This interaction involves the desmin “head” domain and a 24-amino acid motif at the end of the SPRY domain of myospryn. Both proteins colocalize in close relation to the nucleus and the endoplasmic reticulum in neonatal cardiomyocytes as well as at intercalated discs, costameres, and lysosomes in adult cardiac muscle. It has been postulated that the desmin–myospryn interaction plays a role in lysosome biogenesis and positioning [96]. Moreover, desmin has been shown to interact with myotubularin, a protein mutated in X-linked centronuclear myopathy (XLCNM or myotubular myopathy). XLCNM-causing mutations of myotubularin were found to interfere with the desmin–myotubularin interaction resulting in an abnormal desmin IF network in skeletal muscle. Furthermore, downregulation as well as expression of mutant myotubularin both induced morphological and functional defects of mitochondria [82]. In myometrial cells, desmin has been found to directly interact with surfactant protein A (SP-A), a member of the collectin family of proteins. This interaction has been reported to inhibit the polymerization of desmin IFs [51].

#### Interactions with sarcomeric and membrane-associated proteins

Desmin has been reported to directly interact with components of the myofibrillar apparatus. The M-line protein myomesin-1 (synonym: skelemin) was first described as a desmin binding partner in striated muscle [143]. In addition, the coiled-coil rod domain of desmin interacts with the Z-disc section of nebulin [8]. Smooth muscle basic calponin, a major actin-, tropomyosin-, and calmodulin-binding protein, has been identified as a third sarcomeric desmin binding partner, which also interacts with the desmin rod domain [50].

The desmin IF network of muscle cells is anchored to the subsarcolemmal cytoskeleton of costameres. In this respect, desmin has been reported to bind to red blood cell spectrin and it has been postulated that non-red blood cell spectrins also may mediate the association of the desmin IF network with the plasma membrane [101]. A further link between the desmin IF network and the subsarcolemmal cytoskeleton is provided by the direct interaction of an N-terminal region of the desmin head domain with ankyrin [54]. Moreover, desmin was found to directly or indirectly interact with the nicotinic acetylcholine receptor, where it has a postulated role in the submembranous organization of the motor end plate [117].

#### Interactions with small heat shock proteins, apoptosis-related proteins, and nucleic acids

The two small heat shock proteins HspB1 (synonyms: Hsp25, Hsp27) and HspB5 (synonym: αB-crystallin) directly bind to desmin [19, 91]. The interaction of HspB1 with desmin depends of the phosphorylation of HspB1 at serine residue 15 [91]. Furthermore, desmin has been identified as a substrate of caspase-6 and calpains. During the process of apoptosis, caspase-6 cleaves human desmin at the conserved asparagine 264 (corresponds to mouse desmin Asp263) located in the L12 linker (Fig. 4). The N-terminal desmin cleavage product has been considered to play a role in the execution of apoptosis via a dominant-negative effect on the desmin IF network integrity [32]. Desmin also is a specific target of the proteolytic calpain (Ca^2+^-activated cysteine proteinase) system [60]. The limited proteolysis of desmin by calpains results in desmin “head,” “rod,” and “tail” domain cleavage products, which are no longer capable to participate in desmin IF formation, but instead heavily interfere with the proper assembly process [15, 17, 122]. The calpain system has also been attributed to exert a role in the spatiotemporal regulation of desmin protein levels during myotube formation [44]. Like other IF proteins, the head domain of desmin has been demonstrated to bind to single-stranded RNA and DNA molecules in vitro [188, 192]. This property reflects the high proportion of basic residues (12 arginines) and the absence of negatively charged residues in the non-α-helical “head” domain. The in vitro interaction of desmin and DNA is preferentially targeted to exposed single-stranded sites of repetitive and mobile DNA sequence motifs and seems to induce changes of the DNA configuration [181].

## Pathophysiology

### Subcellular localization and expression of wild-type and mutant desmin

**Fig. 7:**
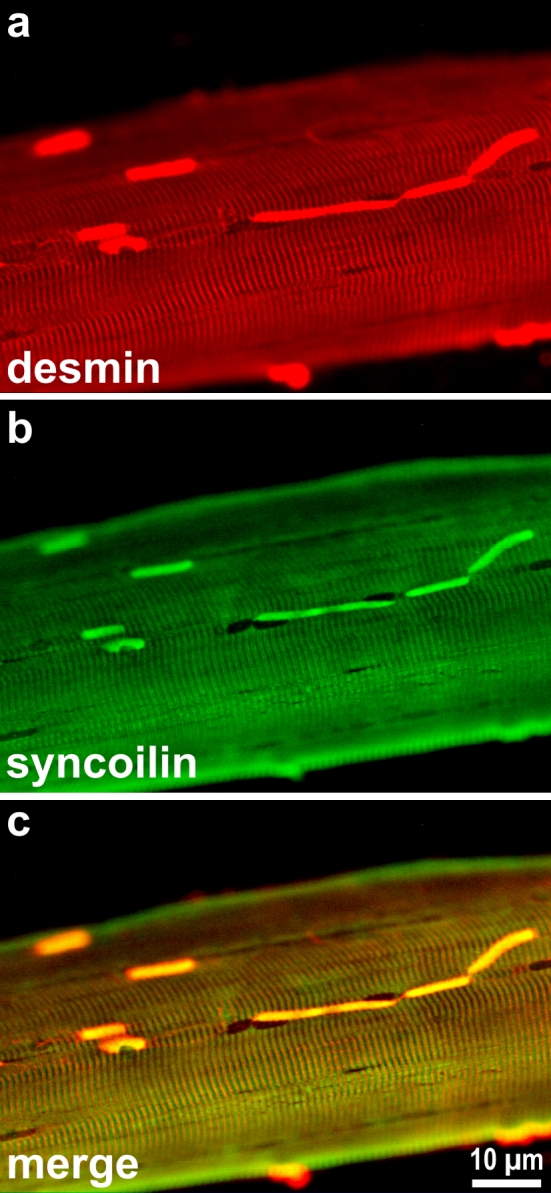
Indirect immunofluorescence labeling of desmin and syncoilin in an isolated muscle fiber from a desminopathy. Note the presence of multiple pathological protein aggregates in addition to the normal cross-striated staining pattern of these two proteins

Analysis of single skeletal muscle fibers from a patient with the heterozygous p.Arg350Pro desmin mutation showed the presence of pathological desmin-positive protein aggregates in conjunction with the normal cross-striated desmin staining pattern [158, 159] (Fig. 7). This finding raises the question whether mutant desmin forms mixed desmin IFs or mixed aggregates with wild-type desmin or if the mutant protein segregates from the wild-type protein. This issue cannot be solved using commercially available desmin antibodies as they do not distinguish between the wild-type and the point-mutated desmin protein species.

Studies on desmin protein expression in desminopathies due to deletion or frame-shift mutations would allow at least the determination of the expression level of the mutant desmin species. However, only two studies reported such data yet. In one study immunoblotting revealed two desmin bands in cardiac and skeletal muscle with the lower band corresponding to a truncated desmin protein species showing an intensity of approximately 30 % of the upper wild-type band. However, no desmin mutation analysis was reported [3]. In another study on a desminopathy due to a heterozygous c.735G>C mutation, desmin immunoblotting revealed the presence of two bands at the positions of 53 kDa and 50 kDa. However, RT-PCR analysis in this particular family identified three distinct desmin mRNA species coding for wild-type and p.Glu245Asp desmin, which both are visible at 53 kDa, as well as p.Asp214_Glu245del corresponding to the truncated 50-kDa protein. Here, the amount of the truncated desmin protein varied between 5 and 43 % [35]. Two-dimensional desmin gel electrophoresis in desminopathies revealed non-uniform results. In a desminopathy due to the heterozygous p.Lys240del mutation, an aberrant and more acidic spectrum of desmin spots was visible [158, 159], whereas the p.Asp214_Glu245del desmin mutant species presented with a more alkaline pI [35].

### Aberrant modifications of desmin

Desmin has been found to be oxidated and nitrated in muscle fibers containing pathological protein aggregates. These modifications may have a direct cytotoxic effect and may impair the degradation via the ubiquitin-proteasom system [87]. Beyond specific desmin modifications, skeletal muscle samples from desminopathies exhibited increased levels of glycoxidated, lipoxidated, and nitrated proteins [88].

### Dysfunctions in protein quality control

A study demonstrated an increased immunoreactivity of the 20S proteasome core as well as its 19S and 11S (synonyms: PA28alpha/beta, PSME1/PSME2) regulators co-localizing with pathological protein aggregates in desminopathies. Further components of the immunoproteasome have also been reported to colocalize with the protein aggregates [46]. Another study demonstrated an enrichment and co-localization of the mutant ubiquitin UBB+1 as well as the autophagy-related p62 with desmin-positive protein aggregates in desminopathies [131]. Desmin-positive protein aggregates in desminopathies also show a positive immunoreactivity with antibodies directed against HspB1 (synonyms: Hsp25, Hsp27) and HspB5 (synonym: αB-crystallin) [160]. With regard to HspB1, two-dimensional gel electrophoresis demonstrated a shift of the main HspB1 spot to a more alkaline pI [36]. Further relationships between the expression of mutant desmin and the ubiquitin–proteasome system, autophagy, and heat shock proteins are provided in the respective paragraphs on desmin mouse models.

## Mitochondrial pathology

SDH and COX stains of skeletal muscle biopsy specimens from desminopathy patients often show areas with either increased or decreased enzyme activities (Fig. 1c). With regard to a putative mitochondrial pathology, an analysis of the mitochondrial function in isolated saponin-permeablized skeletal muscle fibres from a desminopathy patient (heterozygous p.Lys240del desmin mutation) revealed an in vivo inhibition of complex I activity [158, 159].

### Cytoskeletal organization

Desmin transfection studies have been performed in a variety of muscle and non-muscle cell types: BHK21 (ATCC CCL-10) hamster kidney fibroblasts express desmin and vimentin intermediate filaments [80]. HL-1 cardiac [34], C2C12 (ATCC CRL-1772), and inducible C2.7 [141] skeletal muscle-derived mouse myoblasts, human coronary artery smooth muscle (hcasmc), and neonatal rat cardiac ventricular myocytes (nrcm) [178] as well as C3H/10T1/2 (ATCC CCL-226) mouse fibroblasts ectopically expressing Myf5 or MyoD all contain desmin and vimentin IFs. 3T3 (ATCC CRL-1658) mouse fibroblasts contain vimentin. Vimentin-free mouse embryonic fibroblasts (MEFs) isolated from vimentin knockout mice and spontaneously immortalized do not express cytoplasmic IF proteins [37]. Moreover, both MCF7 (ATCC HTB-22) human epithelial cells and bovine mammary gland epithelium cells grown in the presence of hormones BMGE+H [154] are vimentin-free and express only keratin IF proteins. SW13 (ATCC CCL-105) human epithelial cells do not express any IF protein.

**Fig. 8:**
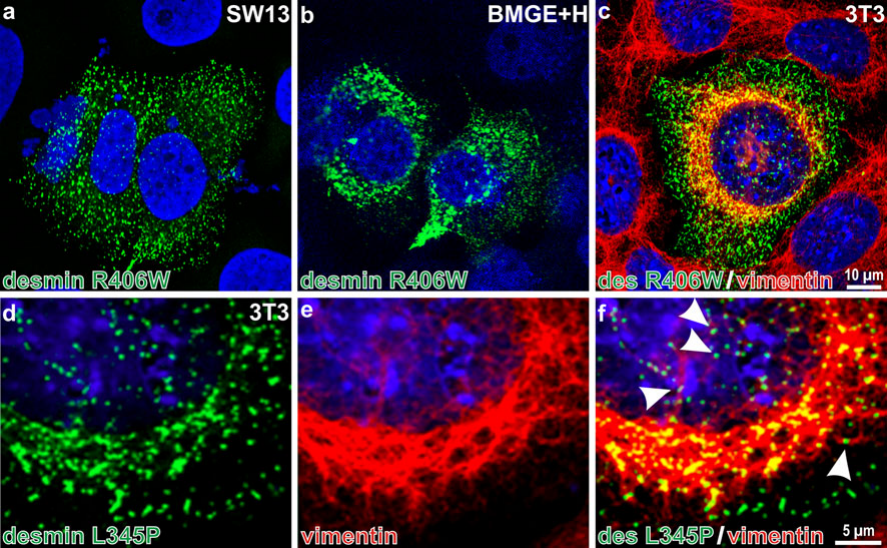
Ectopic expression of mutant desmin in cultured cells. The desmin mutant p.Arg406Trp was transfected into (**a**) IF-free human SW13 cells; **b** vimentin- and desmin-free bovine mammary gland cells (BMGE+H) containing cytokeratins; and **c** into mouse fibroblast 3T3 cells. Detection was by specific primary and secondary antibodies: desmin stain in *green*, vimentin in *red*. Note that the p.Arg406Trp desmin mutant does not form filaments on its own, and also in the vimentin-containing cell the desmin mutant protein stays particulate. Note furthermore that the vimentin system is re-organized (“collapsed”) around the nucleus as a consequence of the mutant desmin expression. In contrast to wild-type desmin, the mutant does not integrate into vimentin filaments [11]. Similarly, the desmin mutant p.Leu345Pro (proline is often called a “helix breaker”) segregates from the endogenous vimentin system when transfected into 3T3 cells: **d** desmin antibody staining; **e** vimentin antibody staining; **f** merged image highlighting *green* dot-like desmin structures on *red* vimentin filaments (*arrowheads*). Also in areas where both proteins are present, they clearly are in distinct structures. Images are taken from [11]

The overall data derived from studies using these cell types indicated that the majority of desmin mutants are incapable of forming a de novo desmin IF network, but instead form non-IF structures and desmin-positive protein aggregates. In addition, most of them induce the collapse of a pre-existing IF network such as in 3T3 cells, although several mutant desmin proteins integrate well, just like the wild-type protein, into the vimentin network [9, 11, 16, 167]. As an example, we depict here the transfection of the p.Arg406Trp desmin rod mutant into various cell types. In the vimentin-free SW13 and BMGE+H cells, it forms dot-like and short rod-like structures (Fig. 8a, b); in addition, in 3T3 cells it completely segregates from the endogenous vimentin filaments and causes the reorganization of vimentin IFs around the nucleus (Fig. 8c). Similarly, the p.Leu345Pro desmin mutant completely avoids integrating into the vimentin system (Fig. 8d–f); however, some affinity for vimentin is observed in extended vimentin IFs below the cell nucleus and outside of the area of the collapsed vimentin IFs (Fig. 8f, arrowheads denote green desmin dots aligning with the red vimentin IFs). In the collapsed area, the green and the red signals superimpose to yield a yellow signal; however, the signals differ entirely in shape, dots versus fibers, indicating that they are not contained within the same structure but are only located close to each other and thereby generate a yellow signal.

### In vitro filament assembly

**Fig. 9:**
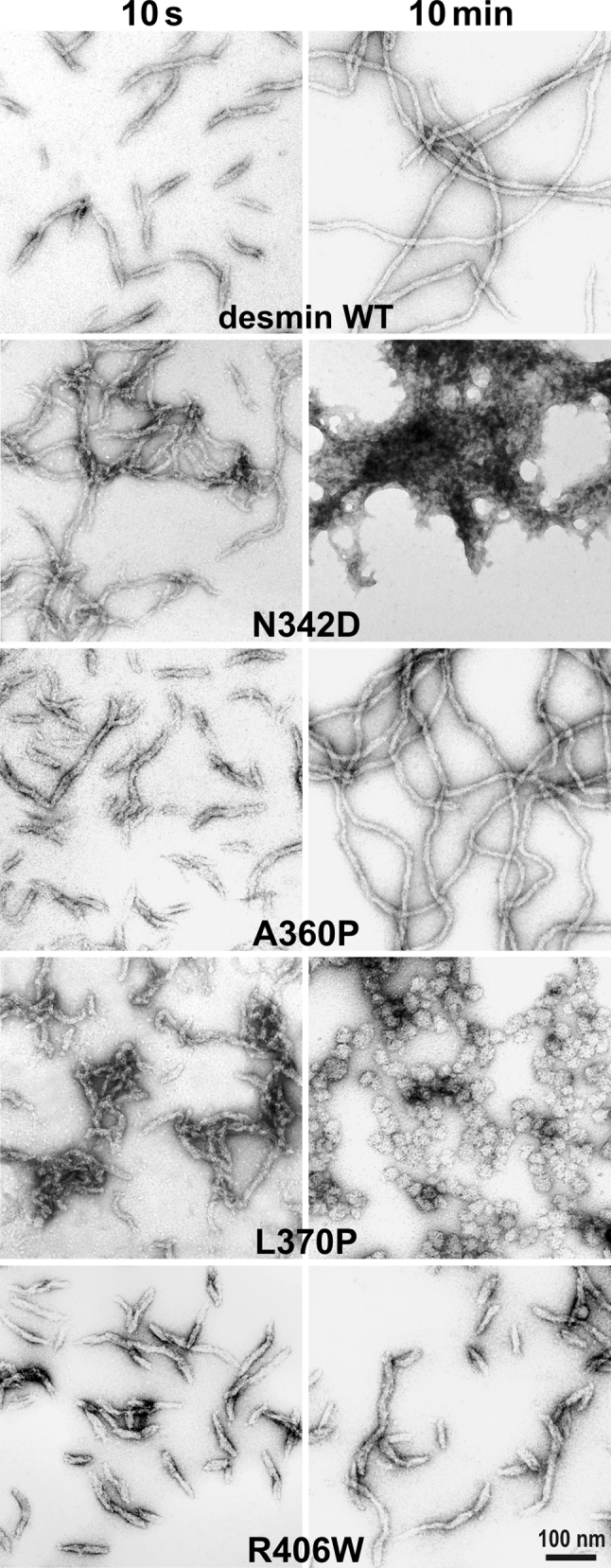
In vitro filament assembly of wild-type desmin compared to various mutants. Assembly was initiated at time point zero by increase of the ionic strength of the incubation buffer and stopped at 10 s and 10 min, respectively, by addition of filament buffer containing 0.2 % glutaraldehyde. The wild-type desmin (desmin WT) and the mutants, indicated within the respective panels, were analyzed after being negatively stained with uranyl acetate by transmission electron microscopy. Images are taken from [12, 13]

The first disease mutant desmin to be analyzed at the in vitro level for its assembly properties was the rather drastic p.Arg173_Glu179del desmin “rod” mutation, which misses seven amino acids in a row in coil 1B. In both cell culture after cDNA-transfection and after forced assembly of recombinant proteins, only non-IF structures were obtained [121]. Later on, several other desmin disease mutants were studied, and their ability to form IF networks in vivo was analyzed by cellular cDNA transfection [11]. In order to reveal how desmin mutations influence basic filament assembly properties, a consecutive study investigated their assembly properties at the protein level [12]. Four major classes of pathological effects were observed (Fig. 9): (1) after 10 s of assembly, filaments form by lateral association of tetramers and subsequent longitudinal annealing of ULFs (see also Fig. 5); however, upon further incubation these IF-like filaments show an abnormal lateral annealing leading to the formation of large “sheets” (Fig. 9, p.Asn342Asp); (2) a second class forms IFs similar to those generated from desmin WT, although the filaments are less regular (Fig. 9, p.Ala360Pro); (3) other mutants exhibit extended filaments at 10 s of assembly, but thereafter immediately decay into ball-like aggregates [13] (Fig. 9, p.Leu370Pro); (4) filament assembly starts with apparently normal ULFs; however, they fail to longitudinally anneal as observed with wild-type desmin and instead stay at the ULF-state or loosely associate longitudinally such that the sub-filament structure remains visible (Fig. 9, p.Arg406Trp). Notably, all desmin disease mutants that were not able to form stable filaments on their own in vitro also did not form IFs after transfection into IF-free cells. Instead, they did segregate from the endogenous IF system when transfected into vimentin- or desmin-containing cells [13]. While the finding that truncated desmin mutants did not properly assemble is not that unexpected, the observed high pathogenicity of missense mutations suggests that desmin filament assembly is a delicate process that can be easily distorted.

### Biomechanics: from filaments to cells and muscle tissue

A characteristic biophysical property of IFs is their elasticity. In response to mechanical stretch, desmin filaments have been shown to extend in length in conjunction with a thinning of their diameter. Experiments demonstrated that upon a 3.4-fold extension, desmin filaments reduced their diameter from 12.6 to 3.5 nm [98]. Moreover, the phenomenon that IFs become more viscoelastic when exposed to an external mechanical force is referred to as “strain stiffening” [86, 155]. In striated muscle, the elasticity of desmin filaments has been attributed to play a cell-protective role against mechanical stress. Thus, desmin filaments may dissipate mechanical energy during muscle contraction.

In this context, mutant desmin may exert a pathogenic effect on the viscoelastic properties of the desmin filament system, which consecutively may lead to progressive muscle fiber damage and muscle weakness. The viscoelastic properties of several disease-associated mutant desmin protein species have been analyzed. Here, the focus was on desmin mutants that are able to form apparently normal filament networks in vitro and in vivo. Some of these mutants showed nanomechanical properties similar to that of wild-type desmin, while others displayed changes in their tensile properties with reduced strain stiffening. Such an increase in the resistance of desmin filaments to an external pulling force may inflict changes in the adaptability of muscle cells to mechanosensing and mechanotransduction [14, 97]. Analysis of primary cultured myoblasts derived from a patient with a heterozygous p.Arg350Pro desmin mutation revealed an aberrant response to mechanical stress. These cells displayed increased cell stiffness and a higher rate of cell death and substrate detachment [22]. A number of biomechanical studies on mice lacking desmin have been reported. One study provided evidence that the lack of desmin is associated with a reduction of the overall passive elasticity of intact isolated soleus muscle [2]. Another study focusing on the diaphragm reported a reduced stiffness and viscoelasticity of this muscle together with an increased tetanic force production [23]. A further study showed a reduced passive tensile strength after eccentric contraction associated with disrupted Z-discs in individual myofibrils. In addition, the maximal isometric force production in the extensor digitorum longus muscle was reported to be decreased, which points to a possible involvement of desmin in excitation–contraction coupling [149]. This assumption was further underlined by an additional study reporting a defective excitation–contraction coupling in intact extensor digitorum longus and soleus muscles of desmin knockout mice. Here, the increased fatigue in extensor digitorum longus muscle was assessed by continuous high frequency field stimulation and could be overcome by the addition of caffeine. This combination of results argues against a defect in the sarcoplasmic reticulum calcium storage capacity [197]. However, this interpretation was challenged by yet another study, which used a more physiological high frequency stimulation protocol with titanic trains. In this setting, a reduced fatigue in intact soleus muscle without any change in the calcium sensitivity of the contractile apparatus was observed [7]. Finally, a study described that the absence of desmin resulted in an increased number of branched myofibers in the flexor digitorum brevis muscle. This work reported neither differences in the resting sarcoplasmic calcium concentration nor in the sarcoplasmic calcium release amplitude in desmin knockout myofibers [61].

## Animal models

### Desmin knockout models

#### *Des*^*ko* *#1*^ and *Des*^*ko* *#2*^

Desmin knockout mouse models from two independent research groups were published in 1996 (*Des* ^*ko* *#1*^ [104] and *Des* ^*ko* *#2*^ [116]). Desmin knockout mice were viable and fertile and showed no overt defects in muscle development. However, they developed clinical and morphological signs of a progressive myopathy and cardiomyopathy [68, 104, 116]. The skeletal muscle pathology, most prominent in weight-bearing muscles, was characterized by disorganized and nonaligned fibers, Z-disc streaming, and subsarcolemmal accumulation of mitochondria. In addition, myofibrillogenesis in regenerating muscles was reported to be disturbed [104, 105, 116]. An in situ analysis of mitochondrial function revealed a reduced maximal rate of ADP-stimulated oxygen consumption [114]. Furthermore, an abnormal folding of the postsynaptic apparatus of the neuromuscular junction was noted [1]. The lack of desmin led to changes in the subcellular distribution of synemin and nestin (synonym: paranemin), but not of plectin [29].

Heart function of desmin knockout mice was investigated by a cardiac MRI study. This demonstrated significantly reduced left and right ventricular ejection fractions and cardiac output, an increased left ventricular mass, and segmental wall thinning and akinesia [171]. In vivo electrophysiological studies revealed reduced atrial but prolonged ventricular refractory periods, ventricular conduction slowing, enhanced inducibility of atrial fibrillation, and a reduced susceptibility to ventricular arrhythmias [157]. Histopathological analyses of cardiac tissue showed areas of hemorrhage, fibrosis, ischemia, and calcification [104, 116]. Isolated cardiomyocytes showed an increased cell volume [115]. In addition, changes in the morphology of intercalated discs, disruptions of the sarcolemma, as well as an abnormal shape and distribution of mitochondria were observed [179]. The mitochondrial pathology in cardiac tissue was further characterized by an aberrant conventional kinesin (synonym: kinesin-1) distribution, a lower amount of cytochrome *c*, and a re-localization of Bcl-2 [109]. Furthermore, ketone body and acetate metabolism, NADH shuttle proteins, amino-acid metabolism, and respiratory enzymes were affected [49].

#### *Des*^*ko* *#2*^/*Bcl*-*2*^*tg overexpression*^

The issue of cardiac pathology was further addressed by crossbreeding desmin knockout mice with transgenic mice overexpressing apoptosis regulator Bcl-2 [196]. These double-mutant mice showed a reduced occurrence of fibrotic lesions in the myocardium, prevention of cardiac hypertrophy, restoration of cardiomyocyte ultrastructure, and significant improvement of cardiac function. In addition, an improved calcium handling of mitochondria was observed. Hence, it was concluded that the mitochondrial abnormalities, which are regarded as the primary cause of the cardiomyopathy in desmin knockout mice, can be ameliorated by the overexpression of Bcl-2.

#### *Des*^*ko* *#2*^/*Des*^*tg p.Ile451Met*^

In an additional study the desmin knockout strain was crossbred with another desmin mouse model with transgenic expression of the p.Ile451Met mutant [113]. Note that only mice homozygous for the desmin knockout and with presence of the p.Ile451Met desmin transgene were analyzed. In this setting, the p.Ile451Met desmin showed an aberrant subcellular distribution, was expressed at lower levels (compared to wild-type desmin in wild-type control mice), and was found to lack the N-terminal 20–30 amino acids. The latter finding has been attributed to a specific cleavage of the mutant desmin in cardiomyocytes.

#### *Des*^*ko* *#2*^/*Des*^*tg p.Asp263Glu*^/TNF^*tg overexpression*^

In the context of a mouse model for tumor necrosis factor alpha-induced cardiomyopathy, it was found that increased expression of TNF-alpha leads to degradation and changes in the subcellular distribution of desmin and to pathological aggregate formation. The latter process was attenuated in TNF-alpha overexpressing mice that additionally expressed p.Asp263Glu mutant desmin instead of the wild-type endogenous protein in the heart. This effect has been attributed to a resistance of the mutant desmin to caspase-6 cleavage [134].

### Desmin transgenic mouse models

#### *Des*^*tg truncated/chimeric desmin*^

In 1996 the first transgenic desmin mouse was published [145]. In this model a hamster desmin–vimentin chimeric protein (last 129 aa of desmin replaced by last 13 aa of vimentin) was expressed under control of the hamster desmin promoter. The expression of this truncated desmin (approximately 10 % mutant compared to wild-type desmin) had a dominant-negative effect on the desmin IF network. Desmin immunostains of skeletal and cardiac muscle tissue revealed an aberrant desmin distribution characterized by a loss of the cross-striated pattern. Ultrastructural analysis showed intermyofibrillar deposits of fibrillar and membranous electron-dense material and fragmented sarcomeres as well as abnormalities of the T-tubule system.

#### *Des*^*tg p.Arg173_Glu179del*^

A second transgenic desmin mouse model expressing a desmin mutant with a small in-frame deletion of seven amino acids was reported in 2001 [194] recapitulating the human mutation reported by [121]. A three-fold overexpression of the p.Arg173_Glu179del desmin compared to the endogenous wild-type desmin led to a dominant negative effect with a disruption of the extra-sarcomeric cytoskeleton and presence of desmin-positive granular filamentous protein aggregates in cardiac tissue. These animals further showed a compromised ability of the heart to respond to β-agonist stimulation. Control animals with a three-fold overexpression of wild-type desmin showed no overt clinical or morphological effect. Skeletal muscle tissue was not addressed in this study.

Electrophysiological investigations of this mouse model showed increased P-wave duration and slowing of ventricular conduction [53]. Although no changes in the expression level of connexin-43, desmoplakin, plakoglobin, and N-cadherin were observed, immunostains showed significantly reduced signal intensities of these junctional proteins. At the ultrastructural level, intercalated discs were found to be highly convoluted and to contain fewer gap junctions and desmosomes.

#### *Des*^*tg p.Arg173_Glu179del*^/Cryab^*tg p.ArgR120Gly*^

Cardiac pathology of the p.Arg173_Glu179del desmin mouse was also analyzed after crossbreeding with transgenic mice overexpressing the p.ArgR120Gly mutant small heat shock protein αB-crystallin [193]. This αB-crystallin mutant had previously been shown to cause a cardiomyopathy characterized by desmin-positive protein aggregates in heterozygous humans and transgenic mice [187, 195]. The double-mutant mice presented with an accentuated cardiac pathology and died of congestive heart failure in the first 2 months of life. Further analyses revealed increased levels of desmin proteins and an increased abundance of pathological aggregates compared to p.Arg173_Glu179del desmin mice.

#### *Des*^*tg p.Arg173_Glu179del*^/GFPdgn^*tg*^

The p.Arg173_Glu179del desmin mouse was further crossbred with a transgenic mouse model expressing the GFPdgn ubiquitin–proteasome system reporter substrate (GFP fused to an ubiquitination signal sequence) [100]. This study provided evidence that the p.Arg173_Glu179del mutant desmin impairs the proteolytic function of the ubiquitin-proteasome system by an impaired entry of ubiquitinated proteins into the 20S proteasome [110].

#### *Des*^*tg p.Arg173_Glu179del*^/GFP-LC3^*tg*^

Moreover, the p.Arg173_Glu179del desmin mouse had been crossed with a autophagy reporter mouse model expressing a GFP-LC3 fusion protein [120]. Here, expression of the desmin deletion mutant led to an increased autophagic flux associated with an increased expression of p62 [200].

#### *Des*^*tg p.Leu345Pro*^

The only transgenic mouse model expressing a desmin missense mutation in addition to the endogenous protein was reported in 2008. The animal model with low expression (approximately 10 % of total desmin) of the HA-tagged p.Leu345Pro desmin mutant was reported to show a reduced contractile function and recovery from fatigue in soleus muscle. Moreover, cardiac alterations consisting of a hypertrophic left ventricular posterior wall and decreased left ventricular chamber dimension were described. Though no MFM typical desmin-positive protein aggregates were detected, this animal presented evidence for a mitochondrial pathology characterized by swelling and vacuolization as well as increased calcium levels [94].

## Conclusion and outlook

The last 2 decades have led to a number of important insights into the molecular pathogenesis of desminopathies, but the precise and sequential molecular mechanisms leading from mutant desmin to consecutive pathological protein aggregation and progressive muscle damage still remain to be elucidated. Though many desmin mutants obviously compromise the desmin filament formation, the progressive human muscle pathology cannot solely be attributed to such a simple mechanistic explanation. If desmin mutants have such a toxic effect on the desmin filament system, why does it take decades of life until clinical symptoms of muscle weakness become apparent? Our review of the currently available data on desmin and desminopathies enforces a complex and multilevel concept of disease development in which mutant desmin additionally interferes with the binding to desmin interaction partners, signaling cascades, protein quality control systems, and the function of cell organelles. Further work is needed to evaluate, broaden, and integrate these different aspects. Respective future studies shall provide essential novel insights into the atomic structure of the desmin tetramer and its assembly reactions, the biomechanics of desmin-mutant cells and tissues, the expression and subcellular localization of mutant desmin, the composition of pathological protein aggregates, the characterization of aberrant post-translational modifications and interactions of desmin with other proteins and nucleic acids, epigenetic factors, and dysfunction of various cell organelles. For studies of early disease stages, the generation and characterization of physiological cell and mouse models, i.e., with a knockin of a desmin mutation into the murine desmin gene, are necessary.

To date, no specific treatment is available for desminopathies and even basic clinical questions—e.g., is physical exercise beneficial or harmful for the course of the disease?—cannot finally be answered on the basis of the currently available literature. A regular neurological, cardiological, and pulmonological diagnostic workup is certainly mandatory for all desminopathy patients. Future studies on desminopathy-related cell and animal models will provide answers with respect to potential therapies: Do anti-aggregation drugs such as small heat shock protein inducers and autophagy and proteasome modulators have a cell-protective effect that ameliorates or even cures the progressive muscle pathology? Or should we further explore muscle-specific gene transfer approaches, for example, siRNA-based silencing of the mutant desmin allele, overexpression of the wild-type desmin, or expression of other protective proteins such as heat shock proteins and Bcl-2?

## Abbreviations

APEG1: Aortic preferentially expressed protein 1 (synonym: SPEG)
BAG-3: BAG family molecular chaperone regulator 3
BLOC-1: Biogenesis of lysosome-related organelles complex 1
CCD: Cardiac conduction defects
CHPF: Chondroitin sulfate synthase 2
CK: Kreatine kinase
COX: Cytochome *c* oxidase
DES: Desmin gene
DNAJB6: DnaJ homolog subfamily B member 6 (synonym: heat shock protein J2)
ECG: Electrocardiogram
EMG: Electromyography
FHL1: Four and a half LIM domains protein 1
GFAP: Glial fibrillary acidic protein
GT: Gomori trichrome
H&E: Hematoxylin and eosin
Hsp: Heat shock protein
IF: Intermediate filament
LCR: 5′ locus control region
MEF: Mouse embryonic fibroblast
MFM: Myofibrillar myopathy
mrf4: Muscle-specific regulatory factor 4 (synonym: myf-6)
MRI: Magnetic resonance imaging
myf-6: Myogenic factor 6 (synonym: mrf4)
myoD: Myoblast determination protein 1
NADH-TR: Reduced nicotinamide adenine dinucleotide tetrazolium reductase
PA28: Proteasome activator 28 (synonym PSME)
PAS: Periodic acid Schiff
PSME: Proteasome activator complex subunit (synonym: PA28)
SDH: Succinic dehydrogenase
SDS-PAGE: Sodium dodecyl sulfate polyacrylamide gel electrophoresis
SHC: Sequence homology class
SPEG: Striated muscle preferentially expressed protein kinase
ULF: Unit length filament
VCP: Valosin containing protein
XLCNM: X-linked centronuclear myopathy
ZASP: Z-band alternatively spliced PDZ-motif protein (synonyms: cypher, LIM domain-binding protein 3)
